# The American System of Dentistry

**Published:** 1886-09

**Authors:** 


### Bibliographical.

THE AMERICAN SYSTEM OF DENTISTRY.—In treatises by various authors. Edited by Wilbur F. Litch, M. D., D. D. S., Professor of Prosthetic Dentistry, Therapeutics and Materia Medica in the Pennsylvania College of Dental Surgery. Published by Lea Brothers & Co., Philadelphia, 1886. This work will be published in three volumes, royal octavo. Price, per volume, cloth, $6 ; leather, $7 ; half morocco, gilt top, $8.

Volume 1st has been issued and includes Regional and Comparative Anatomy, Dental Histology and Dental Pathology. G. V. Black, M. D., D. D. S., Professor of Pathology, Chicago College of Dental Surgery, contributes treatises on "General Pathology," "Dental Caries," "Pathology of the Dental Pulp," "Disease of the Peridential Membrane," and "Abrasion and Erosion of the Teeth." M. H. Cryer, M. D., D. D. S., Chief of Clinic of Oral Surgery, Medico-Chirurgical College, Philadelphia, contributes "Regional Anatomy." James Truman, D. D. S., Professor of Dental Pathology, Therapeutics and Materia Medica, Dental Department University of Pennsylvania, contributes "Diseases of the Dental Pulp and their Treatment." Albert P. Brubaker, A. M., M. D., D. D. S., Professor of Physiology and Pathology, Pennsylvania College Dental Surgery, contributes "Lymphatic Vessels of the Head and Neck." "Embryology and Dental Histology," is contributed by W. Xavier Sudduth, M. D., D. D. S., Demonstrator of Dental Histology, Philadelphia Dental College. Jacob L. Wortman, M. D., Anatomist to the U. S. Army Museum, contributes "The Teeth of the Vertebrates," and W. H. Dall, Curator of the Department of Mollusks, National Museum, Washington, contributes a treatise on "The Teeth of the Invertebrates."

The authors of the above articles are gentlemen well known to the dental profession and recognized as practitioners of distinguished ability. The editor, Dr. W. F. Litch, can congratulate himself on the success of his undertaking as the authors of the articles contained in volume I have certainly treated their subjects in a very able and comprehensive manner.

While many may hold opinions on histology, pathology, &c., and also as to modes of treatment different from those entertained by the authors of the articles in this excellent treatise, all will agree that their work is worthy of commendation and should be greatly appreciated as a valuable contribution to dental literature. While we think it would be best to confine each writer to a definite subject and not have any conflict of opinion on similar subjects in a work of this character, there is no question concerning the value of the views expressed.

As an encyclopœdia of dentistry, for we do not consider the intention was to make it a text-book, on account of its size, expense, &c., it has no superior; besides the style of the greater portion of the text is better adapted to a past graduate course than to the curriculum of a dental school, where comprehensive and abbreviated text-books are necessary for the student entering almost *de novo* into the elements of dentistry. The publishers are to be congratulated on the handsome style of the work, and the material of which the volume is composed. It should form a part of every dentist's library as the information it contains is of the greatest value to all engaged in the practice of dentistry. The remaining volumes will be looked for with great expectation and interest.

---

